# Characteristics of human papillomavirus prevalence and infection patterns among women aged 25–64 according to age groups and cytology results in Ordos City, China

**DOI:** 10.1186/s12985-023-02240-7

**Published:** 2024-01-08

**Authors:** Sumeng Wang, Shujun Liu, Sensen Tan, Jian Yin, Yufei Li, Fanghui Zhao, Youlin Qiao

**Affiliations:** 1 National Cancer Center/National Clinical Research Center for Cancer/Cancer Hospital, Chinese Academy of Medical Sciences and Peking Union Medical College, Beijing, 100021 China; 2 School of Public Health, Chongqing Medical University, Chongqing, 400016 China; 3 Center for Global Health, School of Population Medicine and Public Health, Chinese Academy of Medical Sciences and Peking Union Medical College, Beijing, 100730 China; 4 The State Key Laboratory of Molecular Vaccinology and Molecular Diagnostics, National Institute of Diagnostics and Vaccine Development in Infectious Diseases, School of Public Health, Xiamen University, Xiamen, 361102 Fujian China

**Keywords:** Human papillomavirus (HPV), Genotype, Cervical cancer, Cytology, China

## Abstract

**Background:**

The assessment of human papillomavirus (HPV) genotype distribution in terms of age and cervical lesions could contribute to the adoption of more targeted preventive approaches to specific populations against cervical cancer. The current study was conducted in Ordos City, China, with the aim of analyzing the HPV genotypes prevalence and infection patterns within a hospital-based population.

**Methods:**

The analysis included a total of 26,692 women aged 25–64 who underwent cervical cancer screening between January 1st, 2019, and June 30th, 2022, in Ordos City. These women had valid results for both the polymerase chain reaction (PCR)-reverse dot blot (RDB) HPV test and the liquid-based cytology (thinprep cytologic test/TCT). Data were extracted from the database of KingMed Diagnostics laboratories. The prevalence of HPV genotypes within different age groups and cytology diagnoses were calculated.

**Results:**

Among 26,692 women, 7136 (26.73%) women were HPV positive, 5696 (21.34%) women were high-risk HPV (HR-HPV) positive, and 2102 (7.88%) women had multiple HPV infections. The most frequently detected HPV genotypes were HPV16 (4.72%) and HPV52 (4.15%), ranking as the first and second most prevalent genotypes, respectively. The prevalence of HR-HPV increased with age groups and severity of cervical lesions. Notably, the positive rate of HR-HPV among women aged 35–64 years showed a decreasing trend over the respective years, ranging from 26.00 to 19.70% (P_trend_ < 0.001).

**Conclusion:**

In conclusion, the epidemiology of HPV genotypes partly reflects the effectiveness of regional cervical cancer prevention and control efforts in the past. These findings can inform future initiatives concerning HPV vaccination and screening in the region.

**Supplementary Information:**

The online version contains supplementary material available at 10.1186/s12985-023-02240-7.

## Introduction

Cervical cancer ranks as the fourth leading cause of cancer incidence and death with an estimated 604,000 new cases and 342,000 deaths in women worldwide [1]. In China, cervical cancer has posed a significant burden, as indicated by the rising incidence and mortality rates, with an average annual percentage change (AAPC) of 8.5% and 5.4%, respectively, between 2000 and 2016 [2]. Consequently, effective prevention and control measures for cervical cancer are imperative, yet challenging in China.

Persistent human papillomavirus (HPV) infection has been unequivocally identified as the primary cause of cervical cancer. Fortunately, only a small percentage of HPV infections lead to cytology abnormalities, and among those, only a subset of cervical abnormalities progress to invasive cancer [3]. HPV genotypes have been categorized into low-risk HPV (LR-HPV) and high-risk HPV (HR-HPV) based on their carcinogenic potential. Out of more than 40 HPV genotypes associated with genital infections, HPV16, 18, 31, 33, 35, 39, 45, 51, 52, 56, 58, 59, 66, and 68 have been classified as HR-HPV by the International Agency for Research on Cancer (IARC) [4]. Conversely, other LR-HPV genotypes, such as HPV6, 11, 42, 43, are known to pose a low oncogenic risk and typically cause other genital tract diseases like condylomas.

Cervical cancer preventive methods mainly include HPV vaccination (primary prevention) and screening (secondary prevention). In terms of primary prevention, three types of prophylactic HPV vaccines have been approved for use in many countries, including the bivalent HPV16/18 vaccine, quadrivalent HPV6/11/16/18 vaccine, and nine-valent HPV6/11/16/18/31/33/45/52/58 vaccine [5]. For secondary prevention, HPV testing has been recommended by the World Health Organization (WHO) for primary screening, and more than 254 commercial HPV tests detecting different HPV genotypes or its combination are currently available [6, 7]. The distribution of HPV genotypes can vary regionally on a global scale, including in China [8]. This variation is influenced by factors such as different ethnic groups, living habits, and geographic locations [9]. Therefore, it is important to understand the distribution of HPV genotypes within specific populations, taking into account age groups and the severity of cervical lesions. This knowledge can provide valuable guidance for the implementation of regional HPV vaccination programs and HPV-based screening programs.

Ordos City, situated in northern China, is among the largest cities in Inner Mongolia, with a resident population of approximately 2.2 million [10]. Despite having a vast territory, and a diverse ethnic population, the city has been burdened with a high incidence of cervical cancer and had limited medical resources. While its disease burden has been previously documented, there remains a lack of studies investigating the distribution of HPV genotypes and their correlation with age and cytology results [11]. Therefore, to enhance our knowledge of HPV genotype distribution in this particular region and establish a baseline prevalence of HPV genotypes for monitoring the effectiveness of HPV vaccination and screening in the future, this retrospective cross-sectional study aims to achieve the following objectives: (1) Investigate the characteristics of HPV genotypes distribution among the hospital-based population of Ordos City. (2) Explore the trends of HPV infection over a four-year period in Ordos City. (3) Explore the relationship between HPV genotype distribution concerning age groups and cytological results.

## Materials and methods

### Study population and samples

KingMed Diagnostics, a prominent independent laboratory in China, offers diagnostic testing services to 66 hospitals in Ordos City. Over a period spanning from January 1st, 2019, to June 30th, 2022, a total of 83,675 samples were collected from these hospitals. These samples were primarily obtained from clinical settings to facilitate accurate diagnoses.

Within this dataset, the final analysis included a total of 26,692 women aged 25–64. These women were selected based on having valid HPV testing results (the polymerase chain reaction (PCR)-reverse dot blot (RDB) HPV test) and the presence of concurrent cervical liquid-based cytology diagnoses (thinprep cytologic test/TCT). This subset of individuals formed the basis for further analysis and data evaluation.

### Genotype-specific test and cytology test

DNA extraction and HPV genotyping were performed using Human Papillomavirus Genotyping Kit For 23 Types (PCR-RDB) (Yaneng Bioscience, Shenzhen, China) to detect 14 HR-HPV types and 9 LR-HPV types (HR-HPV: HPV16, 18, 31, 33, 35, 39, 45, 51, 52, 56, 58, 59, 66, and 68; LR-HPV: HPV 6, 11, 42, 43, 81, 53, 73, 82 and 83). The L1 consensus HPV PGMY09/PGMY11 primer set was utilized to amplify the extracted HPV DNA or control within the reaction system. Subsequently, HPV genotyping was performed using hybridization and RDB techniques on strips affixed with 23 distinct type-specific probes. The positive identification of HPV was determined by the presence of visible blue spots on the strip, which were observable without the aid of magnification.

The cervical cytology diagnosis of the study population was divided into four groups based on the severity of the cervical lesions: negative for intraepithelial lesion or malignancy (NILM), atypical squamous cells of undetermined significance (ASCUS), low-grade squamous intraepithelial lesion (LSIL), atypical squamous cells, cannot exclude high-grade squamous intraepithelial lesion and greater (ASC-H+) including ASC-H, atypical glandular cells (AGC), high-grade squamous intraepithelial lesion (HSIL), and squamous cell carcinoma (SCC).

### Statistical analysis

A database was created using Microsoft Excel (version 2021), and the data was analyzed by Microsoft Excel and SAS® statistical software (version 9.4). The prevalence of HPV genotypes was assessed on an annual basis. To examine the trend in HPV infection over the years, chi-square tests for the trend were conducted. Additionally, we calculated the prevalence of HPV genotypes within different age groups and cytology diagnoses based on three criteria: (1) based on the carcinogenic capacity: only HR-HPV infection, only LR-HPV infection, and any HPV infection. (2) based on infection pattern: single HPV infection, multiple HPV infection (infected with two HPV genotypes or more), and only HR-HPV multiple infections. (3) based on genotypes targeted by HPV vaccines: bivalent, nine-valent HR-HPV genotype, and HR-HPV genotypes not covered by vaccines (HPV35, 39, 51, 56, 59, and 68). All statistical tests were two-sided, and *P* values < 0.05 were considered statistically significant.

## Results

### Overall characteristics

**Fig. 1:**
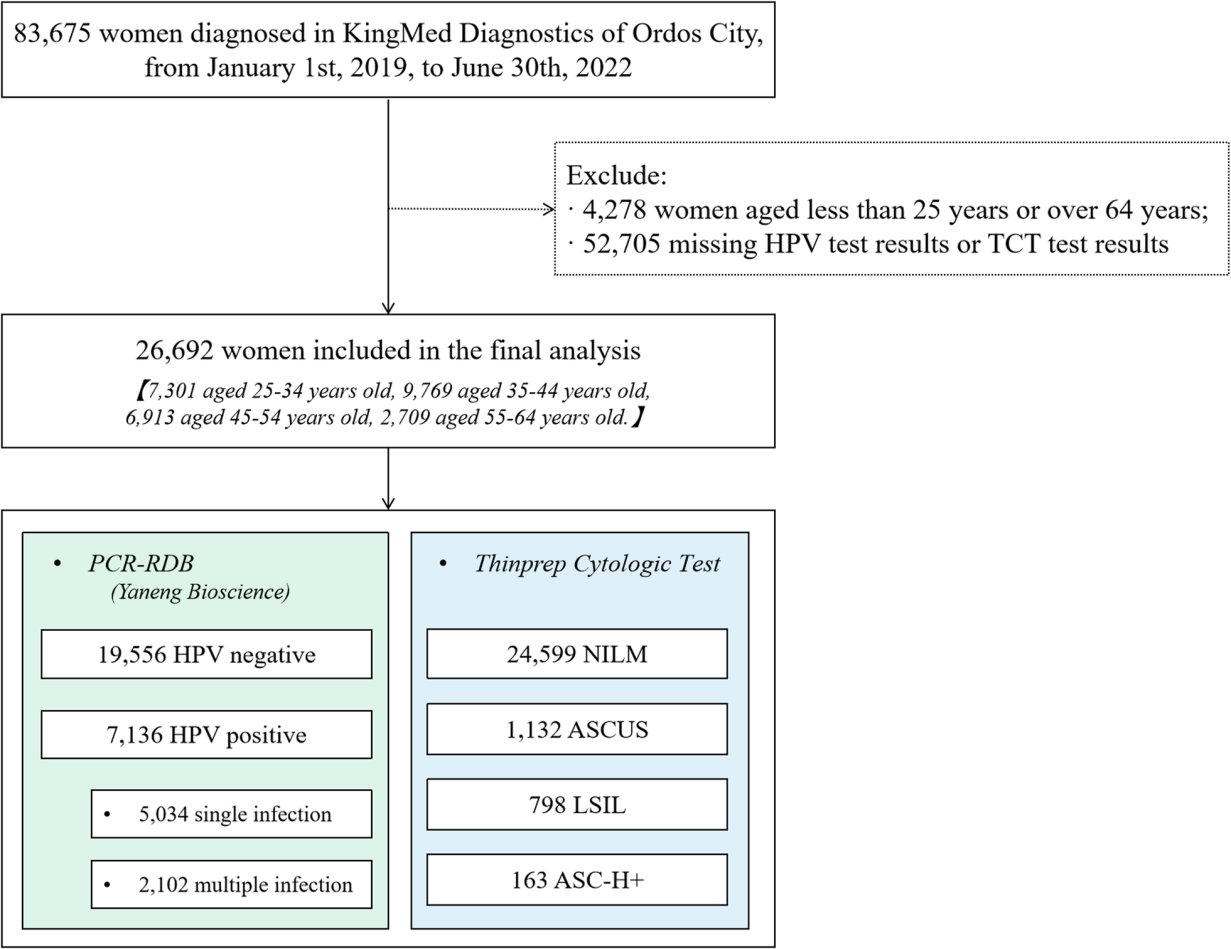
Flowchart of the study population

The study presents the distribution of age groups, cervical cytology results, and types of HPV infection in Table 1 and Fig. 1. In total, 26,692 women were included in the study with a median age of 41 years old (interquartile range: 34–50), of which 27.35% were 25–34 years old, 36.60% were 35–44 years old, 25.90% were 45–54 years old, and 10.15% were 55–64 years old. Approximately 24,599 women had normal cytology results (NILM) and 2093 (7.84%) of women had abnormal cytology results (ASCUS, LSIL, or ASC-H+). A total of 7136 positive samples were detected, resulting in an overall HPV infection rate of 26.73%. Among these, 5696 (21.34%) women tested positive for HR-HPV, 2609 (9.77%) women tested positive for LR-HPV, and 2102 women had multiple infections. The prevalence of HPV genotypes targeted by bivalent, quadrivalent, nine-valent vaccines, and nine-valent (only HR-HPV genotypes) was 5.85% (1561/26,692), 6.81% (1817/26,692), 15.06% (4020/26,692), and 14.31% (3819/26,692) respectively. Additionally, the prevalence of HR-HPV genotypes not targeted by HPV vaccines was 8.68% (2318/26,692).

**Table 1:** Distribution of age, cytology results, types of HPV infection, and HPV genotype distribution prevalence of the study population (N = 26,692)

Characteristics	N	%
Age group (year)		
25-34	7301	27.35
35-44	9769	36.60
45-54	6913	25.90
55-64	2709	10.15
Cytology		
NILM	24,599	92.16
ASCUS	1132	4.24
LSIL	798	2.99
ASC-H+	163	0.61
HPV prevalence (overall)	7136	26.73
Single infection	5034	18.86
Multiple infections	2102	7.88
HR-HPV ^a^	5696	21.34
LR-HPV ^b^	2609	9.77
Vaccination^c^		
Bivalent	1561	5.85
Quadrivalent	1817	6.81
Nine-valent	4020	15.06
Nine-valent (only HR-HPV genotypes)	3819	14.31
Non-vaccine HR-HPV	2318	8.68

^a^HR-HPV: HPV16, 18, 31, 33, 35, 39, 45, 51, 52, 56, 58, 59, 66, and 68;

^b^LR-HPV: HPV 6, 11, 42, 43, 81, 53, 73, 82 and 83;

^c^HPV infection targeted by vaccines

### HR-HPV genotype distribution and predominant genotypes

Among women aged 35–64 years old, approximately 23.36% were found to be positive for HR-HPV. Table 2 presents the positive rates of HR-HPV across different age groups, demonstrating a decreasing trend among women in this particular age range (P_trend_ < 0.001). Additionally, a decline in the prevalence of HPV genotypes 16, 31, 39, 45, 51, 56, 58, and 66 was observed from 2019 to 2022 (all P_trend_ < 0.05). Within the 14 HR-HPV genotypes, the most common types were HPV16 (5.20%), HPV52 (4.40%), and HPV58 (3.14%).

**Table 2:** Distribution of HR-HPV prevalence among women aged 35–64 years old, by year

Characteristics/Year	2019		2020		2021		2022		Total	
	N	%	N	%	N	%	N	%	N	%
Total number	2700	/	5372	/	6502	/	4817	/	19,391	/
HPV16*	160	5.93	275	5.12	351	5.40	223	4.63	1009	5.20
HPV18	39	1.44	88	1.64	83	1.28	54	1.12	264	1.36
HPV31*	53	1.96	76	1.41	109	1.68	55	1.14	293	1.51
HPV33	44	1.63	79	1.47	106	1.63	63	1.31	292	1.51
HPV35	19	0.70	48	0.89	59	0.91	32	0.66	158	0.81
HPV39*	39	1.44	38	0.71	46	0.71	30	0.62	153	0.79
HPV45*	23	0.85	41	0.76	45	0.69	18	0.37	127	0.65
HPV51*	86	3.19	141	2.62	171	2.63	102	2.12	500	2.58
HPV52	115	4.26	233	4.34	314	4.83	192	3.99	854	4.40
HPV56*	85	3.15	142	2.64	184	2.83	95	1.97	506	2.61
HPV58*	99	3.67	166	3.09	220	3.38	124	2.57	609	3.14
HPV59	53	1.96	99	1.84	131	2.01	67	1.39	350	1.80
HPV66*	53	1.96	76	1.41	98	1.51	46	0.95	273	1.41
HPV68	52	1.93	104	1.94	128	1.97	79	1.64	363	1.87
HR-HPV**	702	26.00	1,270	23.64	1,609	24.75	949	19.70	4,530	

^*^P < 0.05; **P < 0.001

**Fig. 2:**
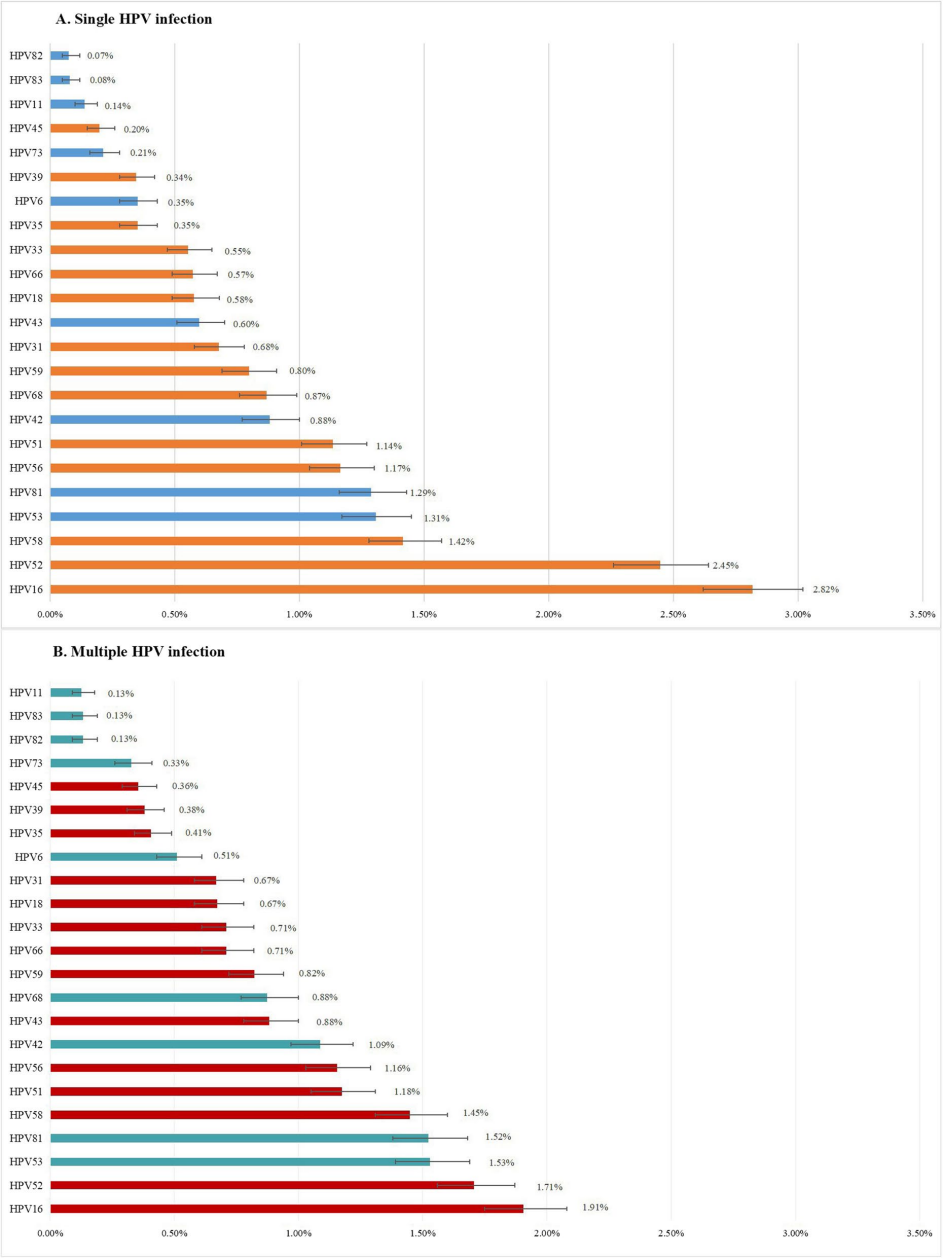
The estimated prevalence of HPV genotypes

Figure 2 displays the distribution of HPV genotypes and the predominant genotypes among the single-infection and multiple-infection groups. Both HPV16 and HPV52 were found to be the most predominant genotypes in both the single HPV infection and multiple HPV infection groups. Multiple HPV infections were found to include all 23 HPV genotypes. The occurrence of different HPV genotypes in multiple infections varied, with the following percentages for each respective genotype: HPV16 (1.99%), HPV52 (1.79%), HPV53 (1.63%), HPV81 (1.58%), HPV58 (1.49%), HPV56 (1.21%), HPV51 (1.19%), HPV42 (1.16%), HPV68 (0.91%), HPV43 (0.90%), HPV59 (0.85%), HPV66 (0.75%), HPV33 (0.74%), HPV31 (0.71%), HPV18 (0.70%), HPV6 (0.55%), HPV35 (0.42%), HPV39 (0.40%), HPV45 (0.36%), HPV73 (0.33%), HPV82 (0.14%), HPV83 (0.14%), and HPV11 (0.13%).

### Epidemiological patterns of HPV genotype infection by age groups and cytology results

**Fig. 3:**
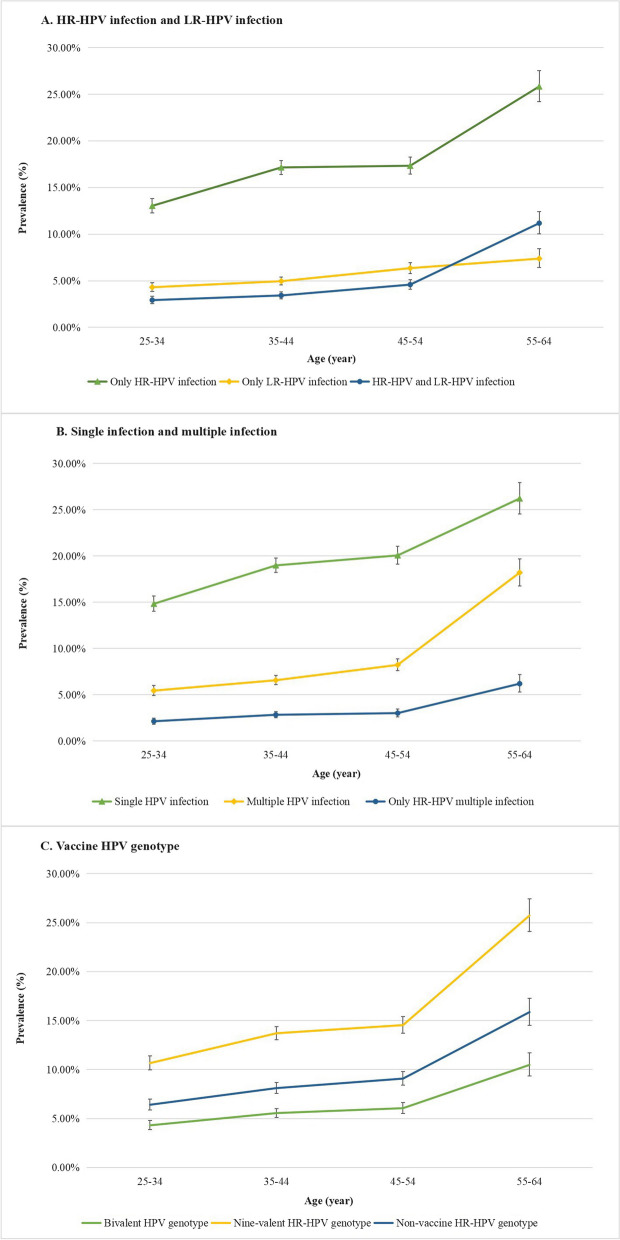
The estimated prevalence of HPV infection by age group. **A** Only HR-HPV infection, only LR-HPV infection, HR-HPV, and LR-HPV infection. **B** Single, multiple infections, and only HR-HPV multiple infections. **C** Bivalent, nonavalent HR-HPV genotype, and non-vaccine HR-HPV genotype

Figure 3 illustrates the estimated prevalence of HPV genotype infection by four age groups (25–34 years old, 35–44 years old, 45–54 years old, and 55–64 years old). Overall, there was an increasing trend in the prevalence of HR-HPV infection with age, ranging from 13.04 to 25.84%. This upward trend was also observed in both single HPV infections (from 4.31 to 7.38%) and multiple HPV infections (from 5.45 to 18.20%). The prevalence of HR-HPV genotypes targeted by the nine-valent HPV vaccines (HPV16/18/31/33/45/52/58) among four age groups were 10.66%, 13.71%, 14.54%, and 25.73%, respectively. For the more detailed age-specific prevalence of HPV genotype infection, please refer to the Additional file 1: Table S1.

**Fig. 4:**
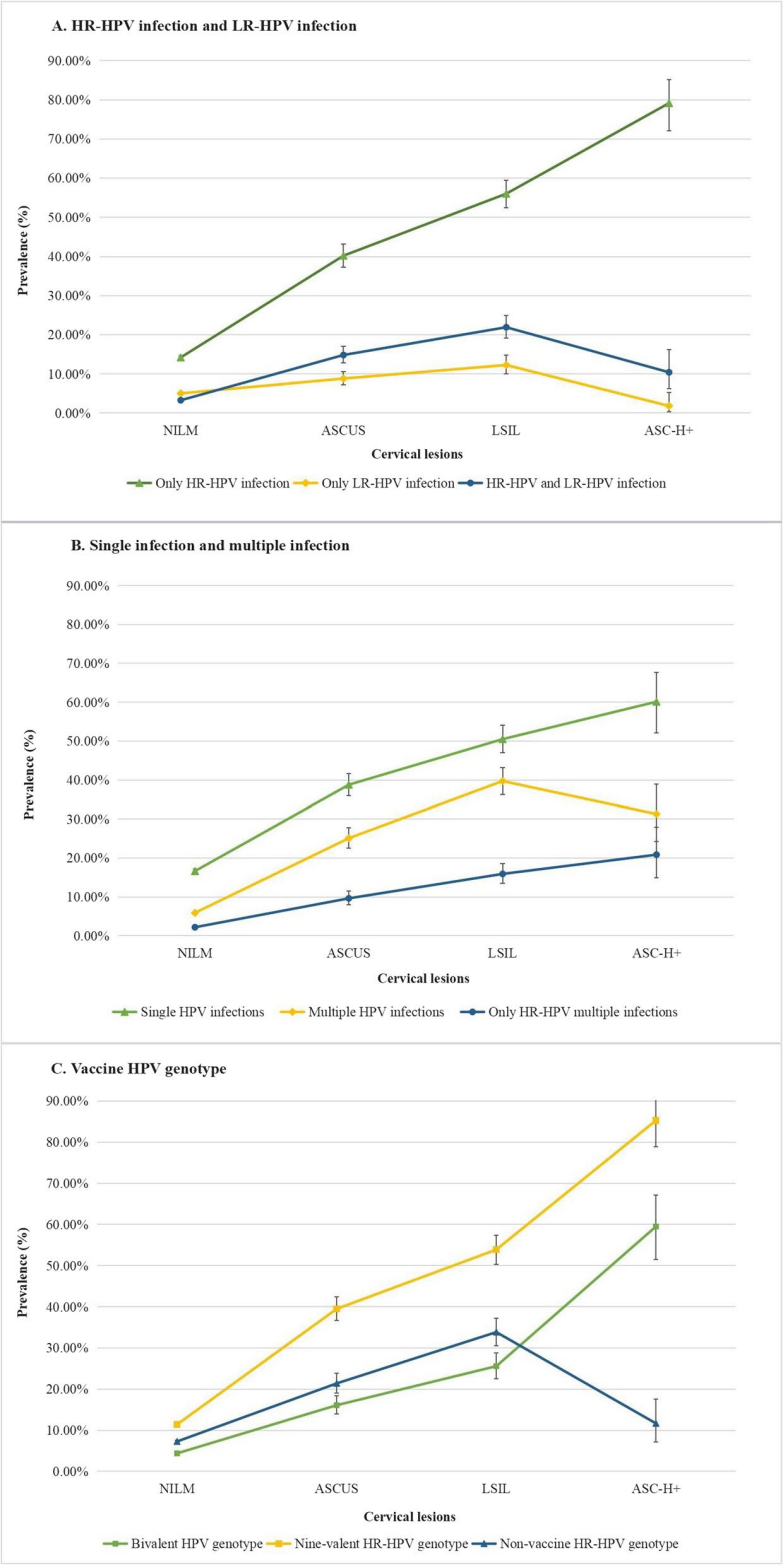
The estimated prevalence of HPV infection by cytological results. **A** Only HR-HPV infection, only LR-HPV infection, HR-HPV, and LR-HPV infection. **B** Single, multiple infections, and only HR-HPV multiple infections. **C** Bivalent, nonavalent HR-HPV genotype, and non-vaccine HR-HPV genotype

Figure 4 depicts the estimated prevalence of HPV genotype infection based on cytology results. Of the 24,599 women diagnosed as NILM, 1132 diagnosed as ASCUS, 798 diagnosed as LSIL, and 163 diagnosed as ASC-H+, the prevalence of HR-HPV infection increased significantly from 14.21 to 79.14%. Conversely, the prevalence of LR-HPV infection initially rose from 5.04 to 12.28% but subsequently decreased to 1.84% in the ASC-H+ group. Both single HPV infection and multiple genotypes infection with only HR-HPV increased with the severity of cervical lesions. Multiple HPV infections showed an increase among the NILM, ASCUS, and LSIL groups, followed by a slight decrease in the ASC-H+ group. The prevalence of HR-HPV genotypes targeted by the nine-valent HPV vaccines (HPV16/18/31/33/45/52/58) among four groups were 11.51%, 39.90%, 54.67%, and 85.08%, respectively. For a detailed presentation of the prevalence of individual HPV genotypes infection among different cytology results, please refer to the Additional file 1: Table S2.

## Discussion

The current study examined the HR-HPV prevalence among women who underwent hospital-based cervical cancer screening in Ordos City, China. The findings revealed that 21.36% of women tested positive for HR-HPV. Moreover, a decreasing trend in HR-HPV prevalence among women aged 35–64 years old was observed from 2019 to 2022. The most frequently detected HPV genotypes were HPV16 and HPV52. The age-specific HR-HPV prevalence exhibited an upward trend, with higher prevalence observed in response to more severe cervical lesions. The epidemiology of HPV genotypes reflects the progress achieved in regional cervical cancer prevention and control efforts. Moreover, the detailed information on HPV genotypes serves as baseline data in Ordos City and can inform the future implementation of HPV vaccination and screening initiatives in the region.

The decline in HR-HPV prevalence among women aged 35–64 years old in recent years can be attributed to the efforts made by the local government in Ordos City. In the past decade, the city implemented cervical cancer screening for women in this age group, starting in 2010, and introduced an HPV-based screening program in 2016 [12]. The HR-HPV positivity rate in the governmental-led screening program was 12.81% from 2016 to 2020 [11]. In August 2020, Ordos City initiated a government-led program to provide free bivalent HPV vaccines to adolescent girls aged 13–18 years old. The program initially started in one administrative division and has gradually expanded its coverage [13]. The comprehensive investigation into HPV genotypes in Ordos City offer valuable baseline data for the development of regional HPV-based screening strategies and provide insights into the ecological changes in HPV following vaccination efforts.

The HR-HPV infection rate in our study was significantly higher compared to women aged 35–64 who participated in organized screening between 2016 and 2020 (23.36% vs. 12.81%) [11]. This phenomenon can be attributed to the fact that our study included a hospital-based population, whereby women may have exhibited symptoms of genital diseases and consequently had a higher risk of HPV prevalence compared to healthy women [14]. While the HPV infection rate may vary geographically across different regions in China [15–17], the HR-HPV prevalence in our study was similar to that reported in other hospital-based studies conducted in China (21.62% vs 21.07%) [18, 19].Several factors, such as economic levels, living habits, and knowledge levels, may contribute to the slight variation observed. It is widely accepted that a majority of HPV infections and associated cancers occur in economically disadvantaged areas [18]. Despite Ordos City having a high economic level, our study found that it still experiences a moderate HPV infection rate (estimated gross regional product (GDP) per capita of ￥256,908 (approximately $36,300) [10]). This occurrence can be attributed to Ordos City being a vast region with a certain proportion of herdsmen and ethnic minorities, mainly Mongolian, who may possess limited knowledge about HPV and endure a higher disease burden [20].

Understanding the distribution of HPV genotypes in a region is crucial for formulating an effective prevention strategy. Asian women exhibit distinct HPV genotypes compared to the global statistics. In addition to HPV16 and 18, HPV52 and 58 are prevalent genotypes within the Asian population [21]. In our study, the most frequently identified HPV genotypes were HPV16, 58, 53, 81, and 56, with a significantly higher prevalence of HPV16, 33, 58, and 52 compared to other genotypes in the ASC-H + category. These dominant HPV genotypes should be carefully considered for future vaccine selection and HPV-based screening programs. The present study revealed a rising trend in age-specific HR-HPV infection. The high prevalence of HR-HPV in older women can be attributed to various controversial factors, such as estrogen deprivation, impaired immune response, and contraceptive usage. Additionally, our study revealed that the prevalence of LR-HPV showed a gradual increase among NILM, ASCUS, and LSIL categories, but decreased in the ASC-H + category. This trend further highlights the lower oncogenic potential of LR-HPV infections.

To the best of our knowledge, this study is the first of its kind conducted in Ordos City, focusing on HPV prevalence and genotype distribution across different age groups and cytology diagnosis. However, it is important to acknowledge several limitations within our study. Firstly, the study was conducted exclusively in hospital settings, involving women who underwent hospital-based cervical cancer screening. Therefore, caution should be exercised when generalizing the results to the broader population. Additionally, our study did not collect sociodemographic information from the participants, which could have provided valuable insights. Furthermore, we did not track the subsequent pathology results of women who exhibited abnormal HPV or cytology findings, which could have provided a more comprehensive understanding of the disease progression.

In summary, this study presents a comprehensive analysis of HPV genotype infection within the hospital-based population of Ordos City. The comprehensive findings in Ordos City provide valuable baseline data of regional HPV-based screening strategies and insights into the ecological changes in HPV post-vaccination efforts.

### Supplementary Information

**Additional file 1**.  **eTable 1.** Prevalence of different HPV genotypes infection by age group. **eTable 2.** Prevalence of different HPV genotypes infection by cytology results.

## Data Availability

The data that support the findings of this study are available from KingMed Diagnostics but restrictions apply to the availability of these data, which were used under license for the current study, and so are not publicly available. Data are however available from the authors upon reasonable request and with permission of KingMed Diagnostics in Ordos City, China.

## Abbreviations

AAPC: Average annual percentage change
AGC: Atypical glandular cells
ASC-H+: Atypical squamous cells, cannot exclude high-grade squamous intraepithelial lesions
ASCUS: Atypical squamous cells of undetermined significance
GDP: Gross domestic product
HR-HPV: High-risk HPV
HSIL: High-grade squamous intraepithelial lesion
HPV: Human papillomavirus
IARC: International Agency for Research on Cancer
LR-HPV: Low-risk HPV
LSIL: Low-grade squamous intraepithelial lesion
NILM: Negative for intraepithelial lesion or malignancy
SCC: Squamous cell carcinoma
WHO: World Health Organization
