# Supplementary material for: Characteristics of human papillomavirus prevalence and infection patterns among women aged 25–64 according to age groups and cytology results in Ordos City, China
Source: Virol J. 2024 Jan 8;21:12. doi: 10.1186/s12985-023-02240-7 (PMC10775550; doi:10.1186/s12985-023-02240-7)
Supplement: Supplementary file 1 — Additional file 1. eTable 1. Prevalence of different HPV genotypes infection by age group. eTable 2. Prevalence of different HPV genotypes infection by cytology results. [file 12985_2023_2240_MOESM1_ESM.docx]

**Supplementary Material**

**eTable 1. Prevalence of different HPV genotypes infection by age group.**

| **HPV genotype/ Age (year)** | **25-34** | | **35-44** | | **45-54** | | **55-64** | |
| --- | --- | --- | --- | --- | --- | --- | --- | --- |
|  | **N** | **%** | **N** | **%** | **N** | **%** | **N** | **%** |
| **HR-HPV** | 1,122 | 15.37 | 1,945 | 19.91 | 1,468 | 21.24 | 972 | 35.88 |
| HPV16 | 252 | 3.45 | 442 | 4.52 | 344 | 4.98 | 223 | 8.23 |
| HPV18 | 70 | 0.96 | 111 | 1.14 | 82 | 1.19 | 71 | 2.62 |
| HPV31 | 67 | 0.92 | 124 | 1.27 | 95 | 1.37 | 74 | 2.73 |
| HPV33 | 46 | 0.63 | 127 | 1.30 | 79 | 1.14 | 86 | 3.17 |
| HPV35 | 45 | 0.62 | 57 | 0.58 | 55 | 0.80 | 46 | 1.70 |
| HPV39 | 41 | 0.56 | 73 | 0.75 | 50 | 0.72 | 30 | 1.11 |
| HPV45 | 21 | 0.29 | 56 | 0.57 | 35 | 0.51 | 36 | 1.33 |
| HPV51 | 117 | 1.60 | 217 | 2.22 | 168 | 2.43 | 115 | 4.25 |
| HPV52 | 255 | 3.49 | 371 | 3.80 | 316 | 4.57 | 167 | 6.16 |
| HPV56 | 114 | 1.56 | 209 | 2.14 | 171 | 2.47 | 126 | 4.65 |
| HPV58 | 156 | 2.14 | 258 | 2.64 | 183 | 2.65 | 168 | 6.20 |
| HPV59 | 83 | 1.14 | 135 | 1.38 | 120 | 1.74 | 95 | 3.51 |
| HPV68 | 103 | 1.41 | 168 | 1.72 | 109 | 1.58 | 86 | 3.17 |
| **LR-HPV** | 581 | 7.96 | 908 | 9.29 | 823 | 11.91 | 550 | 20.30 |
| HPV6 | 51 | 0.70 | 74 | 0.76 | 63 | 0.91 | 43 | 1.59 |
| HPV11 | 24 | 0.33 | 22 | 0.23 | 15 | 0.22 | 10 | 0.37 |
| HPV42 | 88 | 1.21 | 157 | 1.61 | 166 | 2.40 | 115 | 4.25 |
| HPV43 | 75 | 1.03 | 119 | 1.22 | 109 | 1.58 | 93 | 3.43 |
| HPV53 | 149 | 2.04 | 245 | 2.51 | 207 | 2.99 | 157 | 5.80 |
| HPV66 | 70 | 0.96 | 106 | 1.09 | 97 | 1.40 | 70 | 2.58 |
| HPV73 | 39 | 0.53 | 44 | 0.45 | 40 | 0.58 | 22 | 0.81 |
| HPV81 | 142 | 1.94 | 213 | 2.18 | 236 | 3.41 | 160 | 5.91 |
| HPV82 | 10 | 0.14 | 22 | 0.23 | 16 | 0.23 | 8 | 0.30 |
| HPV83 | 14 | 0.19 | 13 | 0.13 | 14 | 0.20 | 16 | 0.59 |

**eTable 2. Prevalence of different HPV genotypes infection by cytology results.**

| **HPV genotype/ Cytology results** | **NILM** | | **ASCUS** | | **LSIL** | | **ASC-H+** | |
| --- | --- | --- | --- | --- | --- | --- | --- | --- |
|  | **N** | **%** | **N** | **%** | **N** | **%** | **N** | **%** |
| **HR-HPV** | 4,305 | 17.50 | 623 | 55.04 | 622 | 77.94 | 146 | 89.57 |
| HPV16 | 852 | 3.46 | 146 | 12.90 | 172 | 21.55 | 91 | 55.83 |
| HPV18 | 239 | 0.97 | 44 | 3.89 | 38 | 4.76 | 13 | 7.98 |
| HPV31 | 260 | 1.06 | 58 | 5.12 | 37 | 4.64 | 5 | 3.07 |
| HPV33 | 220 | 0.89 | 40 | 3.53 | 55 | 6.89 | 23 | 14.11 |
| HPV35 | 149 | 0.61 | 29 | 2.56 | 18 | 2.26 | 7 | 4.29 |
| HPV39 | 147 | 0.60 | 20 | 1.77 | 27 | 3.38 | 0 | 0.00 |
| HPV45 | 112 | 0.46 | 19 | 1.68 | 16 | 2.01 | 1 | 0.61 |
| HPV51 | 463 | 1.88 | 84 | 7.42 | 65 | 8.15 | 5 | 3.07 |
| HPV52 | 861 | 3.50 | 124 | 10.95 | 108 | 13.53 | 16 | 9.82 |
| HPV56 | 458 | 1.86 | 56 | 4.95 | 102 | 12.78 | 4 | 2.45 |
| HPV58 | 546 | 2.22 | 97 | 8.57 | 103 | 12.91 | 19 | 11.66 |
| HPV59 | 344 | 1.40 | 41 | 3.62 | 43 | 5.39 | 5 | 3.07 |
| HPV66 | 230 | 0.93 | 52 | 4.59 | 57 | 7.14 | 4 | 2.45 |
| HPV68 | 374 | 1.52 | 44 | 3.89 | 47 | 5.89 | 1 | 0.61 |
| **LR-HPV** | 2,048 | 8.33 | 268 | 23.67 | 273 | 34.21 | 20 | 12.27 |
| HPV6 | 186 | 0.76 | 17 | 1.50 | 23 | 2.88 | 5 | 3.07 |
| HPV11 | 58 | 0.24 | 7 | 0.62 | 6 | 0.75 | 0 | 0.00 |
| HPV42 | 377 | 1.53 | 68 | 6.01 | 76 | 9.52 | 5 | 3.07 |
| HPV43 | 312 | 1.27 | 42 | 3.71 | 40 | 5.01 | 2 | 1.23 |
| HPV53 | 586 | 2.38 | 81 | 7.16 | 89 | 11.15 | 2 | 1.23 |
| HPV73 | 109 | 0.44 | 19 | 1.68 | 15 | 1.88 | 2 | 1.23 |
| HPV81 | 603 | 2.45 | 70 | 6.18 | 77 | 9.65 | 1 | 0.61 |
| HPV82 | 29 | 0.12 | 11 | 0.97 | 12 | 1.50 | 4 | 2.45 |
| HPV83 | 40 | 0.16 | 8 | 0.71 | 8 | 1.00 | 1 | 0.61 |
